# Clinicopathological predictive factors in the early remission of corticotroph pituitary macroadenomas in a tertiary referral centre

**DOI:** 10.1530/EJE-15-1226

**Published:** 2016-04

**Authors:** Przemysław Witek, Grzegorz Zieliński, Katarzyna Szamotulska, Maria Maksymowicz, Grzegorz Kamiński

**Affiliations:** 1 Department of Endocrinology and Isotope Therapy, Military Institute of Medicine, ul. Szaserów 12804-141, Warsaw, Poland; 2 Department of Neurosurgery, Military Institute of Medicine, ul. Szaserów 12804-141, Warsaw, Poland; 3 Department of Epidemiology and Biostatistics, Institute of Mother and Child, ul. Kasprzaka 17a01-211, Warsaw, Poland; 4 Department of Pathology, M. Sklodowska-Curie Memorial Cancer Centre and Institute of Oncology, ul. Roentgena 502-781, Warsaw, Poland

## Abstract

**Objective:**

Corticotroph macroadenomas are a rare cause of Cushing's disease (CD), but their properties are not well-recognised. The aim of this study was to evaluate the clinical and pathological aspects of corticotroph macroadenomas with particular emphasis on proliferation markers and their associations with the efficacy of surgical treatment.

**Design:**

A prospective cohort study was conducted in a tertiary referral centre in Poland.

**Methods:**

In total, 59 patients with CD (20 macroadenomas and 39 microadenomas) were included in this study. Hormonal and imaging parameters, histopathological and ultrastructural features of the corticotroph tumours and the early surgical outcomes were evaluated.

**Results:**

ACTH and ACTH/cortisol ratios were higher in macroadenomas (*P*<0.001 and *P*=0.002 respectively). Greater tumour volumes were associated with higher Ki-67 and p53 expression (*P*_trend_=0.009 and *P*_trend_=0.024 respectively) and the rates of sparsely granulated adenomas (*P*_trend_=0.036). Immediate postoperative remission and early biochemical remission rates were lower in macroadenomas compared to microadenomas (*P*<0.001). A logistic regression model showed that the immediate postoperative remission or early biochemical remission depended on tumour volume (*P*=0.005 and *P*=0.006 respectively) and invasiveness based on Knosp grades 3 and 4 for macroadenomas and a lack of surgical pseudocapsule for microadenomas (*P*=0.004 and *P*=0.007 respectively).

**Conclusion:**

Corticotroph macroadenomas differ from the more common microadenomas not only in terms of hormonal and imaging characteristics but also in terms of immunohistochemical and ultrastructural features and proliferation markers. The early effectiveness of surgery depends primarily on tumour volume and invasiveness.

## Introduction

Diagnosis and treatment of Cushing's disease (CD) continue to be a challenge for endocrinologists and neurosurgeons, as complications of chronic hypercortisolism lead to an impaired quality of life (1, 2, 3). Most cases of CD are caused by pituitary microadenomas, i.e. tumours with the largest diameter of <10 mm. Approximately 5–15% of adrenocorticotrophic hormone (ACTH)-secreting adenomas are corticotroph macroadenomas, which in some cases are characterised by expansive growth, infiltration and destruction of adjacent structures as well as neuro-ophthalmic symptoms (4, 5, 6, 7).

The available literature contains few reports on retrospective analyses, which mainly concern clinical, hormonal and imaging characteristics associated with pituitary corticotroph macroadenomas (4, 6, 8, 9, 10). They rarely mention histological, immunohistochemical and ultrastructural parameters or proliferation markers assessment (11, 12). These should be of particular importance when the issues of aggressive pituitary adenomas are being discussed and a new clinicopathological classification of pituitary tumours is being proposed (13).

The purpose of this study is to characterise the clinical and pathological aspects of corticotroph macroadenomas compared with microadenomas, with a particular emphasis on proliferation markers, their associations with invasiveness of these tumours and the efficacy of surgical treatment.

## Subjects and methods

### Patient population

The study population consisted of 59 consecutive patients with ACTH-secreting tumours clearly confirmed by magnetic resonance imaging (MRI) of the pituitary. They were hospitalised in the Department of Endocrinology, Military Institute of Medicine in Warsaw, between 2012 and 2015, each having a minimum 6-month follow-up. The patients were operated on by the same neurosurgeon using the same surgical protocol. All patients were informed about the aims and methods of the study and each signed an informed consent. The study protocol was approved by the local Ethics Committee.

### Clinical, endocrine and imaging characteristics

The diagnosis of ACTH-dependent Cushing's syndrome (CS) was made based on standard hormonal criteria: increased urinary-free cortisol (UFC), increased serum cortisol levels at 8.00, loss of cortisol circadian rhythm, increased or detectable plasma ACTH levels at 8.00 and a failure to suppress serum cortisol levels to ≤1.8 μg/dl during an overnight dexamethasone suppression test (ODST; 1 mg at midnight). The pituitary etiology of CS was confirmed based on the serum cortisol or UFC suppression >50% with a high-dose dexamethasone suppression test (HDDST; 2 mg q.i.d. for 4800 h) or a positive result of a corticotrophin-releasing hormone stimulation test (100 μg i.v.) and positive pituitary MRI.

All patients underwent MRI of the pituitary (GE Signa, 1.5 Tesla) before and after i.v. injection of gadolinium (Gd-DTPA). A macroadenoma was defined as a tumour with at least one diameter exceeding 10 mm. The maximum tumour dimension was recorded, and the tumour volume (height×length×width×π/6) was estimated in all patients with macroadenomas. Tumour invasion was evaluated according to Knosp classification (14) for all patients with macroadenomas. In microadenoma cases, the presence of histological pseudocapsule was recorded based on the intraoperative assessment (15).

### Pathological assessment

Histopathological examinations were performed in accordance with the 2004 WHO classification (16). Tissue samples were routinely immunostained for pituitary hormones. Ki-67 (MIB1) labelling index (LI) was graded in three categories: i) <3, ii) 3–10 and iii)>10% positive nuclei. An expression of p53 protein (strongly positive nuclei estimated on 10 high power fields (HPF)) was graded in three categories: i) <5, ii) 5–50 and iii) ≥50% positive cells. The mitotic index was defined as the number of mitoses per 10 HPF on H&E-stained slides. The threshold for ‘proliferative tumours’ was set at a mitotic index of more than 2 mitoses per 10 HPF. Corticotroph adenomas were classified on the basis of commonly accepted ultrastructural features, such as densely granulated (DGCA) or sparsely granulated (SGCA). The Crooke's cell adenoma was classified as a distinct ultrastructural subtype (17).

### Postoperative hormonal evaluation and criteria for remission

Blood samples for serum cortisol were collected from all patients at 0600 h on the first and second postoperative days. Hydrocortisone replacement therapy was started after biochemical confirmation of hypocortisolemia or the development of clinical manifestations of adrenal insufficiency. Following the surgical procedure, all patients were subjected to further postoperative evaluations. An immediate, postoperative remission was recognised when the nadir serum cortisol level taken at 0600 h on the first or second postoperative day was lower than or equal to 2.5 μg/dl. The threshold was set for our centre in a previously published observational study (18). Early biochemical remission was recognised on the basis of hormonal assessments 6 months after pituitary surgery. We used the following criteria for early remission: clinical and biochemical evidence of adrenal insufficiency or, in the case of preserved adrenal function, biochemical evidence of eucortisolemia: UFC, morning serum cortisol and plasma ACTH levels within their respective reference ranges, preserved circadian rhythm of serum cortisol and ODST-induced serum cortisol suppression to ≤1.8 μg/dl.

ACTH levels were assessed using an IRMA (ELSA-ACTH, CIS Bio International, Gif-sur-Yvette Cedex, France). The analytical sensitivity was 2 pg/ml (reference range: 10–60 pg/ml). Serum cortisol concentrations were determined by an electrochemiluminescence immunoassay (Elecsys 2010, Roche Diagnostics). Analytical sensitivity was 0.02 μg/dl (reference range: 6.2–19.4 μg/dl).

### Statistical analysis

Methods of descriptive statistics were used in the statistical analysis. The hypotheses concerning the relationship between two categorical variables were verified using the *χ*^2^ test or *χ*^2^ test for trend, depending on the number of categories. The hypotheses concerning the relationship between one categorical variable and one continuous variable were verified using the non-parametric Mann–Whitney test or Jonckheere–Terpstra test (for trend). Exact variants of tests were applied where necessary. Multiple logistic regression model was estimated to study the relationship between categorical outcome variable and independent variables. The level of significance was set at *P*<0.05. All calculations were made using the statistical software package IBM SPSS version 22.

## Results

### Clinical characteristics

Table 1 presents demographic and clinical characteristics of the study group.

### Imaging characteristics

Preoperative MRI scans identified 39 pituitary microadenomas and 20 pituitary macroadenomas. In the case of macroadenomas, the mean maximum tumour diameter was 24.32±15.43 mm (median: 21.75 mm; range: 11–62), and the mean tumour volume was 12 396.8±27 518.5 mm^3^ (median: 2681.6 mm^3^; range: 221.6–92 960.8). We distinguished two subgroups of macroadenomas depending on MRI-based maximum diameter and volume values. In ten cases (50%), the maximum tumour diameter ranged between 10 and 20 mm, which corresponded to a volume of below 2500 mm^3^. In ten cases (50%), tumour size exceeded 20 mm, with the corresponding tumour volume of ≥2500 mm^3^. As the maximum tumour diameter-based classification closely corresponded to the classification based on tumour volume, we used the tumour volume only in our further analyses.

MRI evidence of suprasellar expansion or optic chiasm compression was found in nine patients (45%). Tumour expansion towards one of the cavernous sinuses was demonstrated in 17 patients (85%), while the remaining three patients (15%) exhibited bilateral tumour expansion. The MRI findings were classified according to Knosp classification. Knosp grade 0 tumours were found in 2 (10%), grade 1 in 4 (20%), grade 2 in 4 (20%), grade 3 in 4 (20%) and grade 4 in 6 cases (30%). For the purpose of subsequent analyses, tumours with no evidence of invasion were considered to be Knosp grade 0–2 macroadenomas (ten cases in total; 50%), while tumours with evidence of invasion were graded as Knosp 3 or 4 (ten cases; 50%).

### Hormonal assessments

Median 8.00 serum cortisol levels in the macro and microadenoma groups were 26.8 and 22.9 μg/dl respectively (*P*=0.370). The two groups did not differ significantly in terms of median midnight serum cortisol levels, which were 17.8 and 16.3 μg/dl respectively (*P*=0.399), nor in terms of median cortisol levels following ODST: 13.9 vs 15.8 μg/dl respectively (*P*=0.672). Plasma ACTH levels in the macroadenoma group were significantly higher at 119.5 vs 65.8 pg/ml in the microadenoma group (*P*<0.001). Likewise, the ratio of morning plasma ACTH levels to serum cortisol levels in the macroadenoma group was significantly higher than in the microadenoma group: 5.0 vs 2.7; *P*=0.002.

Associations between tumour volume and plasma ACTH, serum cortisol and ACTH/cortisol ratios are presented in Table 2.

### Surgical treatment

Transsphenoidal surgery was performed in 18 patients (90%) with pituitary macroadenomas and in all 39 patients with microadenomas. The procedure was used in two patients (10%) having giant macroadenomas. Six macroadenoma patients (30%) and one microadenoma patient (2.6%) required repeat surgery, *P*=0.005. The repeat procedures were more common in the case of larger volume tumours (*P*_trend_=0.002), with two of three patients with giant pituitary adenomas of over 10 000 mm^3^ in volume requiring revision surgery.

Surgical exploration of the pituitary gland allowed for the identification of all macroadenomas, and 37 of the 39 microadenomas visualised earlier via MRI scans (a total of 96.6% in both groups). The presence of surgical pseudocapsules was confirmed in 22 cases (59.5%) from the microadenoma group. It was assumed that from a neurosurgical perspective, a favourable prognosis is related to Knosp grades 0–2 for macroadenomas and the presence of surgical pseudocapsule for microadenomas. Conversely, a less favourable prognosis for macroadenomas was Knosp grades 3 and 4 and for microadenomas an absence of surgical pseudocapsule. According to these criteria, we did not demonstrate a difference between the macro and microadenoma groups in terms of the presence of one of the potentially unfavourable neurosurgical factors – these factors were found in ten macroadenoma cases (50%; Knosp grades 3 and 4) and in 15 microadenoma cases (40.5%; no pseudocapsule) (*P*=0.492).

### Histopathological and ultrastructural assessment results

Positive immunohistochemical findings were detected in 54 patients (91.5%) from the entire study group: in all 20 macroadenoma and in 34 microadenoma patients (87.2%). Normal pituitary tissue was found in four cases (10.3%), and no tissue sample was obtained in one case (2.5%).

Ultrastructural assessment was performed in 52 patients from our group (88.1%): in all 20 cases of macroadenoma and in 32 cases (82.1%) of microadenoma. In seven cases of microadenoma, the tumour size was inadequate to secure enough tissue specimen for ultrastructural examination, and in four cases, ultrastructural evaluation revealed normal, acinar pituitary architecture. In the macroadenoma group, there were 13 cases of DGCA (65.0%) and six cases of SGCA (30.0%) as well as one case of Crooke's cell adenoma (5.0%). In the group of 28 microadenomas, there were 25 cases of DGCA (89.3%) and three cases of SGCA (10.7%). We demonstrated that SGCA rates increased with tumour size (*P*_trend_=0.036).

### Mitotic index

The number of mitotic figures was assessed in all patients with positive histopathological findings (*n*=54; 91.5%). In 34 cases (63.0%), we observed no mitotic figures; in 14 cases (25.9%), there was one division figure/10 HPF; in two cases (3.7%) two figures; in three cases (5.6%; 2 microadenomas and 1 macroadenoma) three figures; and in one case (1.9%; 1 macroadenoma) four mitotic figures. The number of mitotic figures did not differ significantly between the micro and macroadenoma groups (*P*=0.137), even after taking into account the adopted categories of increasing tumour volumes (*P*_trend_=0.157).

### Ki-67 expression

The MIB1 LI was evaluated in 54 (91.5%) of the 59 study group patients: in all of the pituitary macroadenomas and in 34 of 39 microadenomas, i.e. in all the cases with a previously confirmed ACTH-staining pituitary adenoma. We divided the MIB1 LI results into three categories: <3, 3–10 and >10% positive nuclei. An exact test for trend demonstrated that as corticotroph tumour volume increased, there was also an increase in Ki-67 antigen expression (*P*_trend_=0.009).

No association was shown between the adopted MIB1 LI categories and tumour ultrastructure, neither in the entire study group in general (DGCA vs SGCA; *P*_trend_=0.746) nor in the macroadenoma (*P*_trend_=0.732) or microadenoma (*P*=0.562) groups individually.

Repeat surgery rates in the whole study group were higher (borderline statistical significance) with patients in the higher MIB1 LI categories. Repeat surgery rates in the MIB1 LI categories (<3, 3–10 and >10%) for the whole group were 7.7, 25.0 and 33.3 respectively (*P*_trend_=0.076), but no such association was observed in the macroadenoma group (*P*_trend_=1.000).

The proportions of mitotic indices >2/10 HPF in each of the three adopted MIB1 LI categories (<3, 3–10 and >10%) for the whole study group were 7.7, 8.3 and 0.0% respectively. An exact test for trend showed no association between the adopted mitotic index categories and Ki-67 antigen expression in any of the 54 patients (*P*_trend_=1.000). Likewise, no such association was observed either in the macroadenoma group (*P*_trend_=0.400) or in the microadenoma group (*P*_trend_=0.326).

The proportions of Knosp grade 3 or 4 in the macroadenoma group in MIB1 LI categories (<3, 3–10 and >10%) were 45.5, 33.3 and 100.0% respectively. We observed no relationship between the Knosp-based extent of parasellar expansion and Ki-67 antigen expression (*P*_trend_=0.377). It is noteworthy that all patients with MIB1 LI >10% exhibited Knosp grade 4 equivalent to the greatest extent of parasellar expansion.

In the microadenoma group, we observed no association between the presence of surgical pseudocapsules and MIB1 LI. Pseudocapsules were present in 35.7% of microadenomas with MIB1 LI <3% and in 50.0% of those with MIB1 LI 3–10% (*P*=0.653) (there were no microadenomas with MIB1 LI >10%.).

### Expression of p53

Expression of p53 was assessed in 52 patients (88.1%): in all 20 corticotroph macroadenoma patients and in 32 of 39 microadenoma patients (82.1%). We adopted three p53 expression categories: <5, 5–50 and >50% of positive nuclei. An exact test for trend demonstrated that as corticotroph tumour volume increased, so did p53 expression (*P*_trend_=0.024).

Further analyses revealed no relationship between the individual p53 expression categories and i) tumour ultrastructure (DGCA vs SGCA) (either in the entire study group; *P*_trend_=1.000) or in the macroadenoma (*P*_trend_=0.779) and microadenoma (*P*=1.000) groups separately); ii) the number of operations (first time vs repeat) (either in the entire study group (*P*_trend_=1.000) or in the macroadenoma (*P*_trend_=0.772) and microadenoma (*P*=1.000) groups separately); iii) mitotic index (≤2 vs >2 mitoses/10 HPF) (either in the entire study group (*P*_trend_=0.741) or in the macroadenoma (*P*_trend_=1.000) and microadenoma (*P*=1.000) groups separately); iv) Knosp grade (0–2 vs 3 or 4) in the macroadenoma group (*P*_trend_=0.586); and v) surgical pseudocapsule status in the microadenoma group (*P*=0.195).

### Atypical corticotroph adenomas

Based on the 2004 WHO classification, seven cases with MIB1 LI >3% and extensive nuclear staining for p53 (13.5%) were qualified as ‘atypical pituitary adenomas’ whereas the remaining 45 cases (86.5%) were ‘typical pituitary adenomas’. We observed a borderline significant trend towards an increase in the rates of combined proliferation markers (atypical pituitary adenomas) with an increase in pituitary tumour volume (*P*_trend_=0.071).

We also assessed the co-existence of the three adopted MIB1 LI categories (<3, 3–10 and >10%) with extensive nuclear staining for p53 (<5, 5–50 and >50% of strongly positive nuclei). The result of an exact test for trend was borderline significant (*P*_trend_=0.060). We observed that although MIB1 LI <3% was associated with low nuclear staining for p53 <5%, higher MIB1 LI (≥3%) and p53 (≥5%), rates did not show a tendency to co-exist, either in the entire study group or in the macroadenoma and microadenoma subgroups separately.

The proportion of reoperated patients (*n*=7) with a combination of MIB1 LI ≥3% and p53 ≥5% was 42.9%, in comparison to 8.9% of the study group patients who underwent first-time surgery only (*n*=45; *P*=0.014).

### Early results of surgical treatment

The median nadir serum cortisol level on postoperative days 1 or 2 in cases of pituitary macroadenoma was 9.60 μg/dl (interquartile range (IQR) 2.16–40.17), while in cases of microadenoma, it was significantly lower at 1.26 μg/dl (IQR 0.66–2.21) (*P*<0.001). We demonstrated that cortisol levels ≤2.5 μg/dl, indicating immediate post-operative remission, were found in a lower proportion of macroadenomas in comparison with microadenomas: 25.0 vs 76.9% respectively (*P*<0.001). Biochemical evidence of early remission (6 months after surgery) was observed in 30.0% of macroadenomas and 84.6% of microadenomas (*P*<0.001).

We showed that the percentages of nadir cortisol level ≤2.5 μg/dl on postoperative days 1 or 2 decreased proportionally to an increase in tumour volume and were 76.9% for microadenomas, 40.0% for macroadenomas <2500 mm^3^ and 10.0% for macroadenomas ≥2500 mm^3^ (*P*_trend_<0.001). Analogically, early remission rates for adopted tumour size categories were 84.6, 50.0 and 10.0% respectively (*P*_trend_<0.001).

In the macroadenoma group, the early postoperative cortisol levels ≤2.5 μg/dl were observed in five of ten patients (50.0%) from the Knosp 0–2 subgroup and in 0 of ten patients from the Knosp 3 and 4 subgroup (*P*=0.033). Biochemical evidence of early remission 6 months after surgery was detected in 60.0% of patients from the Knosp grades 0–2 group and in none of the Knosp grades 3 and 4 subgroup (*P*=0.011).

Early postoperative serum cortisol levels ≤2.5 μg/dl were observed in 95.5% of encapsulated microadenoma cases in comparison with 60.0% of microadenomas without a surgical pseudocapsule (*P*=0.011). Evidence of early biochemical remission was found in 100.0 and 66.7% respectively (*P*=0.007).

An analysis of the entire study group showed that the presence of a favourable parameter regarding local tumour invasion (Knosp grades 0–2 for macroadenomas or surgical pseudocapsule for microadenomas) was associated with significantly higher rates of immediate postoperative remission compared with the presence of an unfavourable parameter (Knosp grades 3 or 4 or absent surgical pseudocapsule) (81.3 vs 36.0%; *P*<0.001). For early biochemical remission, these rates were 87.5 and 40.0% respectively (*P*<0.001).

With regards to ultrastructure of corticotroph tumours, we demonstrated in the entire group that SGCA were associated with significantly lower rates of immediate remission when compared to those of DGCA (22.2 vs 65.8%; *P*=0.017). For early biochemical remission, these rates were 33.3 and 71.1% respectively (*P*=0.034).

Subsequently, we evaluated the rates of immediate postoperative remission (serum cortisol level ≤2.5 μg/dl) in categories based on proliferation marker levels. A test for trend for Ki-67 LI <3, 3–10 and >10% revealed borderline statistically significant results of 66.7, 58.3 and 0.0% respectively (*P*_trend_=0.090). In terms of the adopted p53 immunoreactivity categories of <5, 5–50 and >50%, we revealed no significance (58.3, 66.7 and 50.0% respectively; *P*_trend_=1.000). For early biochemical remission 6 months after surgery, the rates for the adopted MIB1 LI categories were as follows: 71.8, 66.7 and 0.0% respectively (*P*_trend_=0.079), whereas for the p53 categories: 66.7, 66.7 and 50.0% respectively (*P*_trend_=0.650).

We did not show a relationship between the adopted mitotic index subgroups (≤2 mitoses/10 HPF vs >2 mitoses/10 HPF) and the rates of immediate postoperative remission (62.0 vs 50.0%; *P*=1.000) or early biochemical remission (68.0 vs 50.0%; *P*=0.594) in the whole study group.

Table 3 summarises the described relationships between evaluated clinical, radiographic and pathological parameters and early, biochemical remission of CD in our total study population as well as in the groups of micro and macroadenomas separately.

Subsequently, based on forward stepwise logistic regression with predictive variables from Table 3 (i.e. tumour volume, Knosp classification for macroadenomas and tumour pseudocapsule for microadenomas, ultrastructure and MIB1 LI), we showed that the lack of immediate and early remission significantly depends on tumour volume (*P*_trend_=0.005 and *P*_trend_=0.006 respectively) and the variable describing the invasiveness of corticotroph tumours (*P*=0.004 and *P*=0.007 respectively). This variable combines Knosp classification for macroadenomas and surgical pseudocapsule status for microadenomas. The final models were achieved at step 2 of estimation (Table 4).

## Discussion

ACTH-releasing pituitary macroadenomas are a rare cause of CD. The unselected group of 20 patients presented in this study comprised patients with corticotroph macroadenomas hospitalised in our Department of Endocrinology. The over-representation of macroadenomas in the study group (34%) in comparison with other series (6.6–20.6%) resulted from the fact that our institution is a tertiary referral centre for pituitary tumour surgery. The demographic characteristics of these patients as well as the rates of the main complications of hypercortisolism were typical for CD (5, 6, 7, 11, 12).

Radiographic data from the pituitary macroadenoma cases showed a full range of sizes – from intrasellar tumours to giant macroadenomas with the largest diameter being over 65 mm with evidence of invasion and destruction of adjacent structures. The mean maximum tumour diameter in our study (24.3±15.4 mm) was the largest reported in the literature (8, 19).

Our hormonal assessment findings confirmed earlier reports of significantly higher ACTH secretion by macroadenomas in comparison with microadenomas, with an increase in ACTH secretion accompanying tumour volume increase (6, 10, 11, 12). However, we demonstrated no association between greater tumour volume and morning serum cortisol levels. We did not confirm differences between macro and microadenomas in this respect. Existing reports of serum cortisol levels are inconsistent. Earlier results (6, 11) demonstrated a greater cortisol secretion in macroadenomas. Conversely, a recent retrospective analysis suggested a negative correlation between maximum tumour diameter and morning serum cortisol levels (9).

Our findings seem to confirm that macroadenoma patients exhibit a lower response in terms of cortisol secretion than it would appear from the elevation in ACTH secretion accompanying an increase in tumour volume. Some authors explain this by suggesting a less active form of corticotrophin release by pituitary macroadenomas, associated with impaired transformations of proopiomelanocortin (12, 20, 21).

Some earlier reports indicated a lesser reduction in UFC excretion and serum cortisol levels following HDDST in macroadenomas compared to microadenomas (6, 8, 11, 12). In our study group, the routinely used ODST did not reveal any differences in serum cortisol levels between these two groups of patients. This suggests that higher doses may be necessary to demonstrate a lower sensitivity of macroadenomas to the suppressive effects of dexamethasone.

Transsphenoidal selective adenomectomy is the treatment of choice for CD (2, 7). In the case of the corticotroph macroadenomas, especially those with suprasellar expansion and cavernous sinus infiltration, the use of selective procedures is limited (22). Postoperative remission rates for macroadenomas vary widely depending on the adopted remission criteria, ranging from 12.5 to 67% (4, 6, 8, 9, 12, 23). In our group, the immediate postoperative remission rates were significantly lower in the macroadenoma group in comparison to the microadenoma group (25 and 77% respectively). In total, 6 months after surgery, early remission rates were slightly higher at 30 and 85% respectively. The delayed remission was observed in a single case of pituitary macroadenoma and in three of 39 patients with pituitary microadenomas. In this regards, our findings differed considerably from those by Kakade *et al*. (9). They reported the remission rates at 6 months to be four times higher than those in the immediate postoperative period. It seems that such high rates of delayed remission are difficult to explain and would require analysis of remission criteria and also of the adjuvant treatments used after surgery.

Our study population demonstrated that the rates of both immediate surgical remission and early biochemical remission were associated with macroadenoma size with the best results achieved in tumours of less than 2500 mm^3^ in volume. This tumour volume corresponds to the maximum diameter of 20 mm mentioned in a study by Blevins *et al*. (4), whose analysis focused on smaller tumours with a maximum diameter of 27 mm. This fact may explain such a high reported CD remission rate of 67% which is two times higher than in our group or in other studies. Moreover, the hormonal criteria, which were used to document surgical treatment failure at the time when this article was published, were much less strict than those adopted in our study. Large tumour size as a predictor of persistent CD has also been mentioned by other authors (8, 9).

Apart from tumour volume, an unquestionable affect to the surgical outcome is the cavernous sinus invasion. In our study, the extent of cavernous sinus invasion was not arbitrarily assessed, as was the case in the studies by Blevins & Cannavò (4, 8), but was graded according to Knosp classification. We demonstrated that Knosp grades 3 and 4 macroadenomas were associated with lower rates of immediate postoperative remission in comparison with those of Knosp grades 0–2 tumours. None of Knosp grades 3 and 4 adenomas showed either subnormal cortisol levels in the early postoperative period or evidence of early CD remission 6 months after surgery. Both the tumour size and cavernous sinus invasion can be assessed in a preoperative MRI. Thus, they should be treated as a predictive factor of a poor outcome and discussed with the patients before being eligible for surgical treatment.

Knosp classification has been used in characterising the spread of pituitary macroadenomas. Nonetheless, it seems that microadenomas can also be assessed in terms of local invasion within the pituitary itself. Oldfield & Vortmeyer (15) described the histological pseudocapsule of pituitary tumours. Soon afterwards, excision of encapsulated microadenomas was shown to be associated with high rates of remission (22, 23). In our group of microadenomas, 60% of tumours were qualified as encapsulated adenomas. Their excision was also associated with lower postoperative nadir cortisol levels and higher rates of early biochemical remission.

All our macroadenomas and 90% of microadenomas were histopathologically confirmed to be corticotroph tumours. Next, we assessed the ultrastructure of the corticotroph tumours and the proliferation markers of pituitary adenomas.

With respect to the ultrastructural assessment, we have shown that in the macroadenoma group, the proportion of SGCAs was higher than in the microadenoma group and increased in parallel with tumour volume growth. According to some reports, SGCA subtype is believed to be associated with a more aggressive clinical behaviour (24). In our opinion, however, this relationship is indirect and reflects only a greater volume of the SGCA subtype.

With regards to the assessment of proliferation markers, it can be stated that the Ki-67 nuclear LI is the most commonly evaluated using the MIB1 antibody (11, 25, 26, 27, 28, 29). Kovacs *et al*. (30) considered the Ki-67 LI assessment as the most reliable test to assess pituitary tumour aggression, while suggesting potential Ki-67 LI ranges similar to those used in our study, namely: 1–3% for ‘slow growing pituitary adenomas’ and >10% for ‘aggressive’ pituitary adenomas.

An analysis of our material revealed an association between a gradual increase in tumour volume and a higher Ki-67 expression. Katznelson *et al*. (12) demonstrated higher rates of MIB1 LI >3% in corticotroph macroadenomas in comparison with microadenomas (44 vs 18% respectively), without an analysis of their volumes. However, the demonstrated differences were not significant. Nonetheless, a difference in Ki-67 LI between corticotroph macroadenomas and microadenomas was reported by Losa *et al*. (11).

Our study showed no association between higher Ki-67 rates and cavernous sinus invasion assessed with Knosp classification. What is noteworthy is that the highest MIB1 LI scores of >10% were observed only in the Knosp grade 4 macroadenoma group. Some authors believe that such high Ki-67 LI expression should be sufficient to classify a tumour as an aggressive pituitary adenoma. A MIB1 LI score of >10% is equally typical for pituitary carcinomas (16, 30, 31). However, none of our patients showed evidence of metastases, which is the criterion still considered necessary to diagnose pituitary carcinomas (30). The lack of an obvious association between the extent of cavernous sinus invasion and the enhanced proliferation measured with Ki-67 expression suggests that infiltrative tumour growth involves other, not fully understood, factors.

Additionally, we demonstrated an association between the higher rates of early CD remission and lower Ki-67 expression. This relationship proved to be insignificant after adjusting for pituitary tumour volume. Other authors showed a relationship between higher categories of Ki-67 and recurrence rates of mainly hormonally inactive pituitary tumours (26, 27). However, in these studies, a confounding effect of tumour volume was not taken into account.

According to the 2004 WHO classification, ‘extensive nuclear staining for p53 immunoreactivity’ has become one of the diagnostic criteria of ‘atypical pituitary adenomas’. Literature reports on the role of p53 as a marker of proliferation in pituitary tumours are widely inconsistent. While some articles demonstrated an association between the expression of p53 and the invasiveness of pituitary adenomas (28, 32), others showed no such relationship (27, 29). So far, there has been insufficient data on CD, which is different from other subtypes of pituitary adenomas in that macroadenomas constitute a minority of corticotroph tumours.

In our material, we observed an increase in p53 expression parallel with a greater volume of corticotroph tumours. However, we could not confirm any relationship between the established higher score ranges for p53 immunostaining and Knosp grade for macroadenomas, or a pseudocapsule status for microadenomas. This indicates that the co-existence of other factors is apparently necessary to cause tumour aggressive behaviour. As a transcription factor involved in modulation of DNA damage or induction of apoptosis, the adopted p53 LI cut-off values cannot be expected to be in a simple relationship with the evaluated clinical parameters in corticotroph tumours. Moreover, a lack of uniform ‘immune-positivity’ criteria for p53 LI decidedly confounds both the interpretation of microscopy images and their use in clinical practice.

Another aspect of the pathological evaluation of pituitary tumours is the assessment of mitotic index. Reflecting on its role at this point is important because it has been included as one of the criteria for tumour proliferation in the currently debated clinicopathological classification of pituitary tumours (13). As pituitary adenomas are benign tumours, they are typically characterised by a low number of mitotic figures. As much as 63% of the CD cases from our group showed not a single mitotic figure, and the maximum number of mitotic figures was 4/10 HPF. We did not observe a difference between micro and macroadenomas in terms of the number of mitoses; nor was there any association between surgical outcome and the number of mitotic figures.

There have been few studies on pituitary tumours that analysed the mitotic index as a parameter of proliferation. Additionally, there are discrepancies in the way specific mitotic index values are qualified as ‘high.’ Some authors describe a ‘high’ mitotic index as >1 per 10 HPF (27, 33), while others (13) consider it as being ‘high’ with >2 mitoses/10 HPF. This difference translates into a rate of 11 and 7.4% respectively in our group of corticotroph adenomas.

In light of our results on the clinical and pathological aspects of corticotroph adenomas, we consider the proposed clinicopathological classification (13) as an attempt to systematise the most important features of pituitary tumours. This is of particular importance because the WHO classification fails to meet the current prognostic requirements, especially with respect to ‘atypical pituitary adenomas’. However, the role of this clinicopathological classification system in CD adenomas is somewhat questionable. A vast majority of corticotroph tumours are microadenomas, which qualify for surgical treatment due to their clinical manifestations and complications. At the same time, due to their small size, the proposed classification categorises all of them as ‘non-invasive grade 1’ tumours. This is because invasiveness is evaluated based on cavernous sinus invasion, thus mostly on the basis of preoperative MRI findings. During surgery, however, some of the corticotroph microadenomas reveal features of local invasion, impossible to detect via MRI. These features include the lack of a surgical pseudocapsule or dura mater involvement, whereas other microadenomas have a histological pseudocapsule and exhibit no evidence of invasiveness. This intraoperative evidence of local invasion is a very important characteristic in terms of future prognosis, because due to endocrine aspects, all corticotroph tumours must be efficiently operated upon regardless of their size. As we agree that accurate characterisation of pituitary tumours requires considering the clinical, radiographic and histopathologic aspects collectively, it may be sensible to consider modifying the classification for corticotroph tumours, so as to also include features of local invasion in the case of microadenomas.

Our stepwise logistic regression model suggests that the proposed criteria of corticotroph tumour invasiveness based on Knosp classification in the case of macroadenomas and evidence of local invasion (e.g. surgical pseudocapsule) in microadenomas, combined with tumour volume assessment, will more accurately predict an early surgical outcome of CD, than the assessment of the evaluated proliferation markers. In our paper, we have shown that MIB1 LI and p53 LI have a stronger association with tumour size, rather than directly affecting an early surgical outcome. Having said that, we cannot exclude that other molecular markers, not assessed in this study, are independently associated with efficacy of surgical treatment. We also cannot exclude a possible effect of MIB1 LI and p53 LI on late CD recurrence. This, however, was not evaluated in this study due to the relatively short follow-up.

In conclusion, pituitary corticotroph adenomas, especially macroadenomas, pose a significant challenge to teams of endocrinologists, neurosurgeons and pathologists. To our knowledge, this is the first report presenting an interdisciplinary study on pituitary corticotroph macroadenomas which combines not only hormonal, imaging and surgical parameters but also pathological features including markers of proliferation. Such an interdisciplinary approach fits in with the current discussion on defining the term ‘aggressive’ pituitary adenomas and with the attempt to introduce a clinicopathological classification of these tumours.

## Author contribution statement

P Witek and K Szamotulska designed the study; P Witek, G Zieliński and M Maksymowicz collected the data; P Witek, G Zieliński, M Maksymowicz, K Szamotulska and G Kamiński contributed to data analysis and interpretation; P Witek drafted the manuscript; and all authors contributed to the revision and approval of the final version of the paper.

## Figures and Tables

**Table 1:** Clinical and demographic characteristics of the study group.

	**Macroadenoma** (*n*=20)	**Microadenoma** (*n*=39)	**Total** (*n*=59)
Age (years)			
Mean±s.d.	46.6±14.8	41.4±14.0	43.2±14.4
Median	44	39	42
Range	26–79	17–70	17–79
Females			
*n* (%)	15 (75.0%)	32 (82.1%)	47 (79.7%)
Reoperation			
*n* (%)	6 (30.0%)	1 (2.6%)	7 (11.9%)
BMI (kg/m^2^)			
Mean±s.d.	34.0±8.2	29.9±5.8	31.3±6.9
Median	32.1	28.5	29.6
Range	22.0–49.6	18.0–43.2	18.0–49.6
Hypertension			
*n* (%)	17 (85.0%)	30 (76.9%)	47 (79.7%)
Diabetes			
*n* (%)	6 (30.0%)	12 (30.8%)	18 (30.5%)
Osteoporosis			
*n* (%)	4 (20.0%)	6 (15.4%)	10 (16.9%)

**Table 2:** Associations between the corticotroph tumour volume and plasma ACTH, serum cortisol and ACTH/cortisol ratios.

	**Macro and microadenoma** (*n*=59)	**Macroadenoma** (mm^3^)			**Microadenoma** (*n*=39)	***P*_macro vs micro_**	***P*_trend_**
Total (*n*=20)	≥2500 (*n*=10)	<2500 (*n*=10)
ACTH (pg/ml)							
Mean	104.0	166.4	215.1	117.6	72.1	<0.001	<0.001
s.d.	91.2	130.0	159.8	69.7	34.2		
Median	80.0	119.5	192.0	93.7	65.8		
Range	23.0–563.0	38.0–563.0	41.9–563.0	38.0–265.0	23.0–187.1		
Cortisol^8:00^ (μg/dl)							
Mean	26.2	29.2	33.3	25.1	24.6	0.370	0.352
s.d.	11.9	16.2	21.0	8.9	8.8		
Median	24.2	26.8	26.8	25.8	22.9		
Range	10.9–73.4	10.9–73.4	14.4–73.4	10.9–39.2	13.5–45.5		
Cortisol^0:00^ (μg/dl)							
Mean	18.6	20.1	21.5	18.8	17.8	0.399	0.330
s.d.	7.0	8.5	9.7	7.5	6.1		
Median	16.4	17.8	21.0	15.8	16.3		
Range	9.6–42.8	9.6–42.8	10.1–42.8	9.6–32.7	9.7–35.0		
Cortisol^ODST^ (μg/dl)							
Mean	15.6	14.6	14.3	15.0	16.2	0.672	0.662
s.d.	9.4	8.5	8.6	8.9	10.0		
Median	14.5	13.9	14.1	13.3	15.8		
Range	1.6–40.8	1.6–29.8	1.6–29.8	2.6–27.7	1.6–40.8		
ACTH/cortisol							
Mean	4.1	6.0	6.9	5.2	3.1	0.002	0.001
s.d.	3.0	4.1	4.6	3.6	1.4		
Median	3.3	5.0	5.3	4.6	2.7		
Range	1.2–16.7	1.6–16.7	2.9–16.7	1.6–14.3	1.2–5.8		

**Table 3:** Clinical, radiographic and histopathological parameters and early CD remission 6 months after pituitary surgery – univariate analysis.

	**Macroadenoma**			**Microadenoma**			**Total**		
*n*	Remission (%)	*P* value	*n*	Remission (%)	*P* value	*n*	Remission (%)	*P* value
Total	20	30.0		39	84.6		59	66.1	
Tumour volume			0.141_*t*_						<0.001_*t*_
Microadenoma	–	–		39	84.6		39	84.6	
Macro <2500 mm^3^	10	50.0		–	–		10	50.0	
Macro ≥2500 mm^3^	10	10.0		–	–		10	10.0	
Invasiveness			0.011			0.007			<0.001
Knosp 0–2 (macro-), pseudocapsule (micro-)	10	60.0		22	100.0		32	87.5	
Knosp 3,4 (macro-), no pseudocapsule (micro-)	10	0.0		15	66.7		25	40.0	
Ultrastructure			0.605			0.382			0.034
DGCA	13	38.5		25	88.0		38	71.7	
SGCA	6	16.7		3	66.7		9	33.3	
Ki-67			0.762_*t*_			0.559			0.079_*t*_
<3%	11	27.3		28	89.3		39	71.8	
3–10%	6	50.0		6	83.3		12	66.7	
>10%	3	0.0		0	–		3	0.0	
p53			0.559_*t*_			0.552			0.650_*t*_
<5%	11	27.3		25	84.0		36	66.7	
5–50%	5	20.0		7	100.0		12	66.7	
>50%	4	50.0		0	–		4	50.0	
Atypical adenoma			0.613			1.000			0.682
No	15	26.7		30	86.7		45	66.7	
Yes	5	40.0		2	100.0		7	57.1	
Mitoses per 10 HPF			1.000			1.000			0.594
≤2	18	33.3		32	87.5		50	68.0	
>2	2	0.0		2	100.0		4	50.0	

Subscript *t* denotes *P* value in a test for trend.

**Table 4:** Clinical, radiographic and histopathological parameters and early surgical outcomes – multivariate logistic regression.

**Outcome variable**	**Predictor variables**	***P* value**	**OR (95% CI)**
No immediate postoperative remission (serum cortisol >2.5 μg/dl)	Tumour volume	0.005	8.191 (1.909; 35.141)
Knosp 3 and 4 (macro), no pseudocapsule (micro)	0.004	16.914 (2.519; 113.579)
No early biochemical remission	Tumour volume	0.006	18.738 (2.298; 152.776)
	Knosp 3 and 4 (macro), no pseudocapsule (micro)	0.007	57.546 (3.085; 1073.561)
